# Comparison of Unilateral and Bilateral Transforaminal Epidural Steroid Injections in Unilateral Lumbar Disc Herniation: A Randomized Controlled Trial

**DOI:** 10.3390/jcm14010147

**Published:** 2024-12-30

**Authors:** Hanzade Aybüke Ünal, Ahmet Başarı, Bahir Kayra Özgencil, Güngör Enver Özgencil, Süheyla Karadağ Erkoç

**Affiliations:** 1 Department of Anesthesiology and Reanimation, School of Medicine, Ankara University, 06100 Ankara, Turkey; ozgencilge@gmail.com (G.E.Ö.); suheylakaradag@hotmail.com (S.K.E.); 2 Department of Algology, Kayseri City Hospital, 38080 Kayseri, Turkey; dr.ahmetbasari07@hotmail.com; 3 School of Medicine, TOBB University, 06560 Ankara, Turkey; kayraozgencil1@gmail.com

**Keywords:** degenerative disc disease, disability, disc herniation, lumbar, block

## Abstract

**Objective:** To compare the efficiency of unilateral and bilateral transforaminal epidural steroid injections (TFESI) in patients with unilateral lumbar disc herniation (LDH). **Methods:** In this prospective randomized single-blinded study, patients with unilateral LDH were randomly divided into two groups: A unilateral TFESI group; and a bilateral TFESI group. The severity of pain and disability were assessed with the Numeric Rating Scale (NRS-11) and Oswestry Disability Index (ODI) at baseline, 1 week, 1 month, and 3 months after interventions. Treatment response was defined as ≥50% reduction in the NRS-11 at the 3-month follow-up. Changes in medication consumption at 3 months following the interventions were recorded. This study protocol is registered at ClinicalTrials.gov (NCT06240793). **Results:** A total of 104 patients were included in the study (n = 58 in the unilateral TFESI group and n = 46 in the bilateral TFESI group). The NRS-11, ODI scores and medical treatment consumption did not differ statistically between the groups at 3 months (*p* ˃ 0.05). At 3 months, the rates of patients with a > 50% decrease in NRS-11 scores were 13.8% and 32.6% in the unilateral TFESI group and bilateral TFESI group, respectively. **Conclusions:** Unilateral and bilateral TFESI both decrease pain severity and disability scores to a similar degree, although bilateral TFESI was more effective in reducing pain severity by over 50% in patients with single-level unilateral LDH.

## 1. Introduction

Lumbar disc herniation (LDH) is one of the most common causes of low back pain and radicular pain [1]. It often develops as a result of the degeneration of intervertebral discs (IVDs), while other causes include congenital anomalies, trauma and connective tissue diseases [2]. The degeneration of IVDs—the complex fibrocartilaginous structures that connect adjacent vertebral bodies and facilitate spinal movement—increases with age, with over 80% of IVDs showing degenerative changes in those over the age of 50 years [3,4]. This degeneration induces changes in the ratio of Type I and Type II collagen in the nucleus pulposus and annulus fibrosus, leading to herniation of the disc material. [5]. The herniated disc material then causes mechanical compression and inflammation of the nerve root, resulting in pain [1,6].

In most cases with LDH, the pain can be successfully managed with conservative treatment [2]. Additionally, in certain cases, physical therapy methods such as mobilization can also be considered [4]. Epidural steroid injections may be considered particularly in cases in which conservative methods do not yield improvement within 4–6 weeks [7,8]. Epidural steroid injections act by inhibiting the synthesis and release of the pro-inflammatory substances induced by mechanical compression, and can be administered via interlaminar, transforaminal, or caudal routes. In the transforaminal technique, the solution is administered to the anterior epidural space and especially spreads to the ipsilateral periradicular area. In contrast, in interlaminar epidural steroid injections, the solution crosses the midline and spreads bilaterally but remains in the posterior epidural area [6,9]. Caudal epidural steroid injections, however, are effective only in herniations at the lower lumbar level, and require the administration of high volumes [10].

Radicular pain is an outcome of the chemical stimulation around the nerve root sheath or dorsal root ganglion (DRG). The transforaminal route, which targets the anterior epidural area adjacent to the nerve root, has been reported to be superior to pain palliation than interlaminar injection in patients with symptomatic LDH [6,9,11]. While many studies to date have examined the technique of transforaminal epidural steroid injection (TFESI), intervals between TFESI procedures, and the type and optimal dosage of drugs used in TFESI, there have been none comparing the clinical effects of unilateral and bilateral transforaminal injections in cases with single-level unilateral LDH [12,13,14,15,16,17]. Experimental studies have reported that herniated discs cause a release of inflammatory cytokines, affecting not only the ipsilateral DRG but also the intact contralateral DRG [18,19]. As inflammation is thought to be bilateral in unilateral LDH, Erdine S recommends a bilateral TFESI application in cases of unilateral lumbosacral disc herniation [20], although, in most of the studies, unilateral TFESI were used for the treatment of unilateral LDH [6,7,10]. The present study evaluates the effects of unilateral and bilateral TFESI on pain intensity, functionality, and medication use in cases of unilateral LDH, and determines whether one approach is superior to the other.

## 2. Materials and Methods

### 2.1. Study Design

This prospective randomized single-blinded study was conducted in the Department of Pain Medicine of the Faculty of Medicine of Ankara University after being granted approval by the local ethics committee (2023/267). The study was registered on ClinicalTrials.gov (NCT06240793) and was carried out in full compliance with the principles of the Declaration of Helsinki. Written informed consent was obtained from all participants prior to the study.

### 2.2. Patient Population

Included in the study were patients aged 18–65 years with low back pain and radiating unilateral lower limb pain for at least 3 months, with a diagnosis of unilateral LDH confirmed through clinical evaluation, physical examination, and Magnetic Resonance Imaging (MRI), with a lack of response to conservative treatment such as medical and physical therapy, and with a pain intensity score of ≥4 based on the Numeric Rating Scale-11 (NRS-11). Excluded from the study were those with unstable or uncontrolled psychiatric illnesses, pregnancy, bleeding diathesis, a history of an epidural steroid injection within the last year, history of previous lumbar surgery, allergies to the substances used during the procedure (local anesthetics, steroids, contrast materials), scoliosis, spondylolisthesis, central canal stenosis, compression fracture, previous discitis, sequestered disc or bilateral disc herniation.

### 2.3. Randomization and Blinding

Randomization was performed by Random Allocation Software version 1.0 (Saghaei, M, Iran)—a computer-based random number generator—based on which the patients were assigned to two groups. Patients were included in the randomization process in the order of their application to the study. Those assigned an odd number by the software underwent a unilateral TFESI (n = 64), and those assigned an even number underwent a bilateral TFESI (n = 64). All procedures were performed by a single researcher (H.A.Ü.) and the patients were followed up by another researcher who was blinded to the procedures performed (A.B.).

### 2.4. Treatment Protocols

Unilateral TFESI: Intravenous access and standard monitoring were established in all patients. The level of intervention was determined based on the results of clinical, physical, and MRI examinations. All interventions were performed under the guidance of C-arm fluoroscopy. All patients were placed in the prone position and supported with a pillow under the abdomen to reduce lumbar lordosis. An anterior-posterior (AP) image was obtained to visualize the intervention level. The fluoroscope was adjusted cephalad or caudally to align the superior endplate of the target vertebra. An ipsilateral oblique image (10–30 degrees) was taken to achieve a “Scotty Dog” view. The skin was sterilized with povidone-iodine, and local anesthesia (3 mL of 2% prilocaine) was administered to the skin and subcutaneous tissue. A 20-gauge double-holed transforaminal epidural cannula with a blunt tip (Egemen International^®^, İzmir, Türkiye) was advanced to the chin of the Scotty Dog (adjacent to the pars interarticularis and inferior to the pedicle) under intermittent fluoroscopic guidance. In the lateral view, the needle tip was confirmed to be located in the posterior 1/2 to 1/3 of the neural foramen [21]. After negative aspiration for cerebrospinal fluid and blood, 1–2 mL of contrast medium (350 mgI/mL iohexol) was injected under continuous fluoroscopy guidance. Intravascular, subdural, subarachnoid, and intradiscal spread were ruled out through lateral and AP craniocaudal oblique images. After confirming the contrast spread to the anterior epidural space in the lateral view and the nerve root spread along the pedicle on the oblique AP view, 8 mg dexamethasone (2 mL) was injected. All patients were kept under observation for 1 h following the procedure.

Bilateral TFESI: The TFESI procedure was applied as in the protocol described above. For the bilateral TFESI application, after confirming the accuracy of the contrast spread, a mixture of 1 mL physiological saline and 4 mg of dexamethasone (1 mL) was injected into each foramen at the same level. The patients were similarly observed for 1 h post-procedure.

### 2.5. Data Collection and Assessment

The demographic and clinical characteristics of the patients, including age, sex, body mass index (BMI), comorbidities, pain duration, disc herniation level, and side of LDH were recorded ahead of the procedure. Pain intensity was evaluated using the NRS-11 scale, and functionality was assessed using the Oswestry Disability Index (ODI) at baseline, and at 1 week, 1 month, and 3 months after the procedure. The medications used by the patients, including non-steroidal anti-inflammatory drugs (NSAIDs), gabapentinoids, and opioids, were recorded both at baseline and at 3 months post-procedure. Patients were asked about possible side effects during and after the intervention and at all follow-up visits. A positive response to the treatment was defined as at least a 50% reduction in the NRS-11 score at the 3-month follow-up compared to the baseline. Demographic data, preprocedural and postprocedural data, and treatment outcomes were followed and documented by a blinded researcher (A. B). The primary outcome measure for this study was the change in NRS-11 scores after the interventions. The secondary outcomes included the change in ODI scores, medication consumption, and possible complications at 3 months following the interventions.

### 2.6. Statistical Analyses

Power analysis was performed for sample size estimation using G*Power 3.1 (https://www.psychologie.hhu.de/arbeitsgruppen/allgemeine-psychologie-und-arbeitspsychologie/gpower accessed on 1 May 2023). Since there are no similar studies in the current literature, the calculations were performed using a medium effect size of 0.5 as per Cohen’s guidelines. A total of 128 patients (64 per group) was required, with a power of 0.80 and an α value of 0.05. The data were analyzed using IBM SPSS Statistics (Standard Concurrent User License) (Version 29.0.2.0 Armonk, NY, USA: IBM Corp., Armonk, NY, USA). Descriptive statistics were presented as frequencies (n), percentages (%), mean ± standard deviation, median, minimum, and maximum values. The normality of the NRS and ODI data was assessed via the Shapiro–Wilk normality test; the homogeneity of variances was evaluated with Levene’s test; and the age distribution of the treatment types was assessed with an independent samples *t*-test. Categorical variables related to the different treatment types were compared using Yates’ Chi-square and Fisher’s exact tests. When the Chi-square test results were found to be significant, subgroup analyses were conducted using a Bonferroni-corrected two-proportion z-test. The comparisons of medication use from baseline to the third month were made using the McNemar test. The baseline, first-week, and first- and third-month NRS and ODI values recorded for the different treatment types were evaluated with a Two-way repeated measures analysis of variance, with a Bonferroni correction applied for pairwise comparisons. A *p*-value of <0.05 was considered statistically significant.

## 3. Results

**Figure 1:**
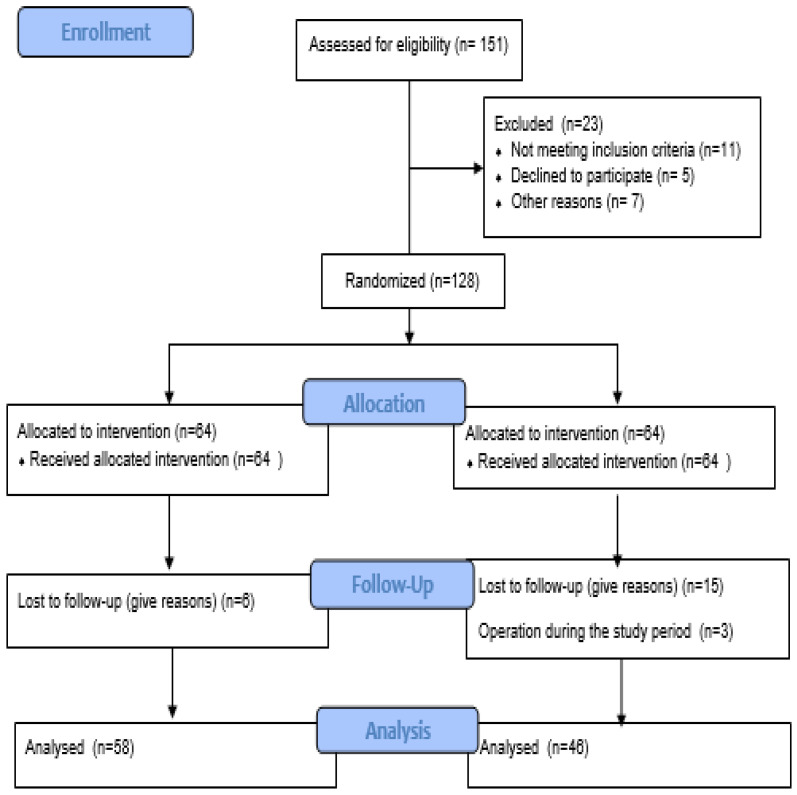
CONSORT diagram.

A total of 104 patients were included in the final analysis (n = 58 in the unilateral TFESI group and n = 46 in the bilateral TFESI group), and the CONSORT of the sample is presented in Figure 1. All of the patients were complained of single level unilateral LDH. The mean age was 47.7 ± 11.1 years. The mean and median duration of symptoms was 0.97 ± 0.53 years and 1.0 (0.32–3.00) years, respectively. None of the patients exhibited any motor deficits. The baseline demographic and clinical characteristics of the patients are presented in Table 1. No significant differences were noted in the demographic or clinical characteristics of the groups (*p* > 0.05).

**Figure 2:**
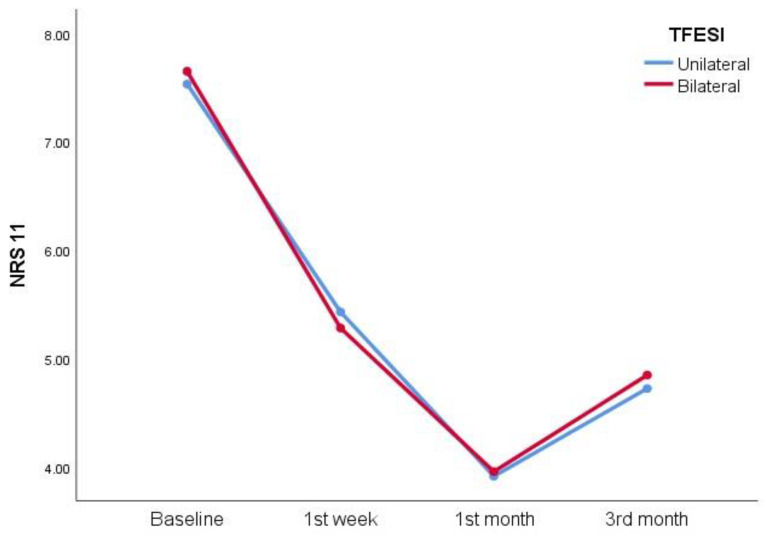
Mean NRS-11 scores at 3-month follow-up.

Both groups achieved a statistically significant improvement in NRS-11 scores at all time points (*p* < 0.001), and the NRS-11 scores were similar between the groups at all follow-up points (Figure 2, Table 2). At 3 months, 13.8% in the unilateral TFESI group and 32.6% in the bilateral TFESI group had achieved a ≥50% decrease in NRS-11 scores. Both groups also showed a statistically significant improvement in mean ODI scores at all time points (*p* < 0.001). In the bilateral TFESI group, the mean ODI scores were significantly lower at 1 week (*p* < 0.05), while similar ODI scores were achieved in the two groups at 1 and 3 months (Table 2).

The rates of NSAID, gabapentinoid, and opioid use at baseline and 3 months post-procedure were not statistically different between the unilateral and bilateral TFESI groups (Table 3). Three cases of temporary paraesthesia last less than 12 h in the bilateral TFESI group. No major adverse events were observed in either group.

## 4. Discussion

We compared the efficacy of bilateral and unilateral TFESI in patients with single-level unilateral LDH. Both methods provided satisfactory results in terms of pain relief and disability over a three-month period. Although there was no difference between the groups in terms of pain intensity and disability, at least a 50% reduction in pain intensity in the 3rd month was more frequently observed in those who underwent bilateral TFESI. In the first week of measurement, the ODI scores were lower in the bilateral group, but this difference did not persist in the subsequent follow-up periods.

In LDH, the mechanical damage caused by the nucleus pulposus (NP) and the chemical mediators released from the disc, trigger an inflammatory response, leading to radicular pain [22]. Epidural injections with corticosteroids aim to inhibit prostaglandin synthesis, stabilize cell membranes, and improve neuronal blood flow, and thus induce the activation of an anti-inflammatory mechanism and radicular pain [23]. The transforaminal epidural approach allows corticosteroids to be delivered directly to the ventral epidural area and DRG, where the primary pathology is located, and this delivery of medication directly to the area of pathology supports greater concentrations of the drug with lower volumes [24]. A recently published meta-analysis evaluated the evidence level of TFESI for radicular pain due to LDH as Level 1, and the same study found TFESI resulted in reduced pain intensity and disability over 3 and 6 months [7]. Similarly, a reduction in pain intensity and disability over 3 months was also noted in the present study following TFESI.

Studies have demonstrated that disc herniation is biologically active and triggers various inflammatory cascades [25]. Inflammatory mediators such as tumor necrosis factor-alpha (TNF-α), interleukin-1 beta (IL-1β), interleukin-4 (IL-4), interleukin-6 (IL-6), interleukin-12 (IL-12), interferon-gamma (IFN-γ), and matrix metalloproteinase (MMP), along with such pain-related factors as nitric oxide (NO) and cyclooxygenase-2 (COX-2), are induced [3,26,27]. Recent studies have also revealed inflammatory markers such as IL-2, IL-6, IL-8, and TNF-α to be significantly elevated in serum samples from patients with LDH [28,29], and the released proinflammatory cytokines lower the pain threshold and increase pain sensitivity [30]. Hiyama A et al. reported radicular pain severity to be associated with certain chemokines released from the disc into the blood serum [30,31]. The aim of epidural steroid administration is to reduce the inflammation caused by herniated discs at the nerve root. The epidural space at the nerve root is known to be connected at the same level on the contralateral side, and in some patients, radicular symptoms on the contralateral side without compression have been attributed to cytokine release from the herniated disc, periradicular fibrosis, and the resultant inflammation [32]. MRI studies have also associated the presence of clinical lumbar radiculopathy without nerve compression but with chemical irritation caused by annular tears [24]. Nakagawa Y et al. identified such signs of inflammation as edema, redness, fibrosis, and adhesions in the contralateral nerve root during the herniated disc during surgery of two patients, despite the presence of compression without inflammation on the ipsilateral side [33]. Animal studies have provided supporting findings related to this condition. It has been reported that DRG neurons activated by peripheral nerve injuries release monocyte chemoattractant protein-1 (MCP-1), and these factors, released by compressed neurons, affect intact DRG. In a neuropathic pain model, an increased expression of the CCR2 ligand and MCP-1 was observed in both compressed and non-compressed DRG neurons [18,34,35]. Brazda V et al. used a sciatic nerve ligation approach on 103 rats to investigate temporal changes in IL-6 and its receptor gp130 in both ipsilateral and contralateral DRGs, and reported increased IL-6 expression not only in the DRG associated with injured nerves, but also in those not associated with nerve injury in an experimental neuropathic pain model [19]. Erdine S. recommended that, despite the unilateral compression in LDH, the inflammation is bilateral, and so bilateral TFESI should be considered in unilateral LDH [20]. Although no studies on this topic have been published to date, it is known that some experts prefer unilateral and others prefer bilateral TFESI when treating unilateral single-level LDHs. Although studies on neuropathic models in animals have shown the spread of inflammation to the bilateral dorsal root ganglia (DRG) [18,34,35], and inflammation has also been detected on the intact contralateral side in humans [33], administering steroids to the bilateral anterior epidural space did not have a significant effect on patients’ pain severity and functionality in this study.

Studies examining the type and dose of steroids used in TFESI have produced conflicting results, particularly regarding the use of particulate versus non-particulate steroids. [36,37]. In a meta-analysis published by Makkar JK et al., particulate steroids are reported to be slightly better at reducing pain than non-particulate steroids [38]. Due to the potentially catastrophic effects associated with particulate steroids, in the present study, we injected dexamethasone in epidural applications, opting for a total of 8 mg dexamethasone in both bilateral and unilateral applications, given the lack of any difference in efficacy between 4 mg and higher doses of dexamethasone in TFESI [39]. In our study, although the total steroid dose used in bilateral and unilateral injections was the same, the difference in injection volume applied to the herniated disc level (2 cc for unilateral and 1 cc for bilateral injections) could raise speculation. Makkar JK et al. reported that selective staining of the nerve root could be achieved with an average of 0.41 mL of contrast in TFESI. They further observed that with higher contrast volumes, the contrast spread superiorly and inferiorly to the targeted epidural area. Additionally, the same study determined that the mean volume at which contralateral spread occurred was 1.46 mL. The fact that we applied 1–2 mL of contrast in our patients supports the notion that the contralateral spread of the medication may have occurred in unilateral applications, which might explain the absence of differences in pain intensity changes [40]. In contrast, Chun EH et al. compared injection volumes of 3 mL and 8 mL with the same diluted steroid dose and found significantly greater pain reduction over four weeks with the higher volume. However, the patient population in their study was heterogeneous, including conditions such as spinal stenosis, spondylolisthesis, and herniated nucleus pulposus. Furthermore, the dose of local anesthetic administered during TFESI differed between patients. Since local anesthetics not only have anti-inflammatory effects but are also believed to suppress ectopic discharges in nerves, the greater effectiveness observed with higher volumes may be attributed to this factor [41]. Furman et al. observed contralateral spread in 52% of cases following a 4.0 mL contrast injection. However, they noted that this contralateral spread rarely resulted in substantial contrast entering the contralateral epidural space. Additionally, when the flow crossed the midline, it rarely completely bathed the contralateral side [42]. In our study, we believe that the medication may have spread contralaterally in some patients due to the 3–4 cc injection volume used in unilateral applications. However, fluoroscopic data to confirm this phenomenon were not recorded in the study.

In the present study, the patients were using NSAIDs, gabapentinoids, or opioids as part of their medical treatment prior to the procedure. There was no significant difference in medication usage in the third month following either method. While studies exist reporting a reduction in opioid use after TFESI [43], to the best of our knowledge, there have been no studies to date comparing the consumption of gabapentinoids, NSAIDs, and opioids post-TFESI. Although the primary aim of this study was to compare the efficacy of bilateral and unilateral TFESI, we also demonstrated a reduction in opioid usage. Although opioid use decreased in the post-treatment period in both groups, the use of gabapentin and NSAIDs increased. Unfortunately, we did not specify these changes in terms of dosage and frequency.

Transforaminal injections can lead to serious complications due to the anatomical proximity of the radicular artery to the nerve root, and can include permanent paraplegia, associated with intra-arterial drug injection, spinal cord infarction, and intradiscal injection [7,12,44,45]. The rate of minor complications in lumbar TFESI procedures ranges between 2.4% and 9.6% [46]. In the present study, transient paraesthesia was observed in three patients in the bilateral TFESI group. The incidence of minor adverse effects was 6.5% in the group that underwent bilateral TFESI, whereas no adverse effects were observed in the unilateral TFESI group. Furthermore, although we did not precisely measure the procedure duration for bilateral and unilateral TFESI, the bilateral TFESI procedure is inherently more time-consuming. As a result, it exposes both patients and healthcare staff to higher levels of radiation.

In patients who underwent bilateral TFESI, the proportion of patients achieving at least a 50% reduction in pain was remarkable. A detailed examination of the patient data revealed that, in the bilateral TFESI group, the percentage of patients with severe baseline pain who experienced pain relief exceeding 50% by the third-month post-injection was significantly higher. However, when NRS scores were analyzed without categorizing baseline values as moderate or severe, no significant difference was observed between the groups during the post-injection period. The statistical difference in achieving at least a 50% reduction in NRS scores between the groups can be attributed to the absence of differences in numerical data but the presence of differences in categorical data. Considering that no significant differences were observed in NRS and ODI scores during the follow-up period and recognizing the risks associated with each additional intervention, we believe the appropriateness of bilateral TFESI for unilateral single-level LDH is debatable. We propose that unilateral TFESI may represent a more reasonable and safer approach.

This study has several limitations, one of which was that the degree of herniation and the types of herniations (foraminal/extraforaminal) were not determined. Although an equal number of patients were distributed among the groups, the number of patients who did not attend follow-up was higher in the group that underwent bilateral TFESI. The selection of patients from a single tertiary center constitutes another limitation of the study. This resulted in the inclusion of a patient population predominantly consisting of individuals with comorbid conditions who were referred to higher-level care facilities. The absence of double-blinding, with patients being aware of the procedure they underwent, could have potentially influenced their responses. Another limitation of the study is that we did not document the prevalence or rate of contralateral spread under fluoroscopy during unilateral TFESI. Furthermore, patient outcomes were evaluated solely based on pain intensity, disability, and medication usage. We did not assess changes in inflammatory markers or cytokine profiles, which are key indicators of inflammation. Lastly, the patients were followed for only 3 months after the injection. Extending the follow-up period would provide valuable insights into potential differences in pain recurrence, if any.

## 5. Conclusions

TFESI is an effective treatment approach in single-level unilateral LDH, due its its ability to reduce pain severity and disability. The primary pain mechanism in LDH is believed to be chemical inflammation affecting the nerve root and DRG. Considering the continuity of the nerve root with the epidural space, it is thought that inflammation may spread to bilateral nerve roots, making bilateral TFESI potentially more effective. In conclusion, bilateral TFESI showed superiority in reducing pain severity by 50% or more at the third month post-procedure; however, no statistically significant differences in pain severity or disability were observed between the two approaches.

## Figures and Tables

**Table 1:** Baseline demographic and clinical characteristics of patients.

Variables	Unilateral TFESI *n* = 58	Bilateral TFESI *n* = 46	*p* Value
Age (yr)	47.4 ± 10.9	48.3 ± 11.4	0.695 ^†^
F/M (n)	39/19	32/14	0.967 ^Փ^
BMI (kg/m^2^)	26.07 ± 2.50	26.17 ± 2.54	0.855 ^†^
Symptom duration, (yr)	0.98 ± 0.51	0.97 ± 0.57	0.903 ^†^
Level of LDH (L3/L4/L5)	3/23/32	3/15/28	0.721 ^¥^
Side of LDH (R/L)	34/24	20/26	0.181 ^Փ^

yr: years, F: female, M: male, n: number, BMI: body mass index, LDH: lumbar disc herniation, R: right, L: left, TFESI: transforaminal epidural steroid injection. Data expressed as numbers or mean ± standard deviation. ^†^: Independent samples *t*-test. ^Փ^: Yates Chi-square test, ^¥^: Fisher-Freeman-Halton exact test.

**Table 2:** Comparison of NRS and ODI scores between groups.

	Unilateral TFESI *n* = 58	Bilateral TFESI *n* = 46	*p Value* ^‡^
**NRS 11**			
Baseline	7.5 ± 1.1	7.6 ± 0.9	0.582
1st week	5.4 ± 1.1	5.3 ± 0.8	0.444
1st month	3.9 ± 0.9	3.9 ± 0.5	0.783
3rd month	4.,7 ± 07	4.8 ± 0.7	0.371
Test statistics ^&^: *F*; *p*	227,247; **<0.001**	199,520; **<0.001**	
**≥50% decrease in NRS-11, n (%)**	8 (13.8)	15 (32.6)	**0.040** ^Ф^
**ODI**			
Baseline	60.2 ± 13.9	57.5 ± 7.5	0.250
1st week	45.0 ± 11.6	40.6 ± 9.7	**0.046**
1st month	30.7 ± 10.4	28.2 ± 6.2	0.167
3rd month	38.7 ± 9.9	36.5 ± 9.0	0.241
Test statistics ^&^: *F*; *p*	204,181; **<0.001**	171,830; **<0.001**	

NRS 11: Numeric Rating Scale 11, ODI: Oswestry Disability Index, TFESI: transforaminal epidural steroid injection n: number. Data are presented as mean ± standard deviation. ^‡^: Comparisons between groups at each measurement time point, ^&^: Comparisons between measurement time points within each group, ^Ф^: Yates’ Chi-square test.

**Table 3:** Comparison of medical treatment consumption between groups.

	Unilateral TFESI n = 58	Bilateral TFESI *n* = 46	*p Value*
**Baseline** *			
NSAID	4 (6.9)	1 (2.2)	0.380 ^ᵵ^
Gabapentinoid	18 (310)	16 (34.8)	0.846 ^Ф^
Opioid	11 (19.0)	8 (17.4)	0.999 ^Ф^
**3rd month** *			
NSAID	14 (24.1)	5 (10.9)	0.138 ^Ф^
Gabapentinoid	49 (84.5)	36 (78.3)	0.575 ^Ф^
Opioid	2 (34)	0 (0.0)	0.502 ^Ф^

n: number NSAID: non-steroidal anti-inflammatory drugs *: A patient may be using multiple medications concurrently. Gabapentinoids include gabapentin and pregabalin. Data are presented as numbers (%), ^ᵵ^: Fisher’s exact test, ^Ф^: Yates Chi-square test.

## Data Availability

Data available upon reasonable request.
